# Improving the diagnosis of hyperphagia in melanocortin‐4 receptor pathway diseases

**DOI:** 10.1002/oby.24287

**Published:** 2025-06-17

**Authors:** M. Jennifer Abuzzahab, Beatrice Dubern, Anthony P. Goldstone, Andrea M. Haqq, Steven B. Heymsfield, Jennifer L. Miller, Jesse Richards, Martin Wabitsch, Jack A. Yanovski

**Affiliations:** ^1^ Diabetes and Endocrine Center Children's Minnesota St Paul Minnesota USA; ^2^ Sorbonne Université, Trousseau Hospital Assistance Publique‐Hôpitaux de Paris Paris France; ^3^ Sorbonne Université, Inserm, Nutrition and Obesities, Systemic Approaches Research Group Paris France; ^4^ PsychoNeuroEndocrinology Research Group, Division of Psychiatry, Department of Brain Sciences, Faculty of Medicine, Imperial College London Hammersmith Hospital London UK; ^5^ Imperial Centre for Endocrinology, Imperial College Healthcare NHS Trust Hammersmith Hospital London UK; ^6^ Division of Pediatric Endocrinology University of Alberta Edmonton Alberta Canada; ^7^ Pennington Biomedical Research Center Louisiana State University System Baton Rouge Louisiana USA; ^8^ Department of Pediatrics University of Florida College of Medicine Gainesville Florida USA; ^9^ Department of Internal Medicine University of Oklahoma at Tulsa Tulsa Oklahoma USA; ^10^ Division of Pediatric Endocrinology and Diabetes, Center for Rare Endocrine Diseases, Department of Pediatrics and Adolescent Medicine University of Ulm Ulm Germany; ^11^ Section on Growth and Obesity, Division of Intramural Research Eunice Kennedy Shriver National Institute of Child Health and Human Development, National Institutes of Health Bethesda Maryland USA

## Abstract

Characteristics of hyperphagia include heightened and prolonged hunger, longer time to satiation, shorter duration of satiety, severe preoccupation with food (i.e., hyperphagic drive), abnormal food‐seeking behaviors, and distress or functional impairment when food is unavailable. Patients with melanocortin‐4 receptor (MC4R) pathway diseases including those caused by variants in one of multiple key genes of the pathway often present with hyperphagia that results in early‐onset, severe obesity because this pathway plays a critical role in regulation of hunger/satiation and energy balance. Patients with syndromic obesity (e.g., Bardet‐Biedl syndrome) may also have hyperphagia as a result of neurodevelopmental disruptions in the MC4R pathway. Genetic testing is suggested in patients with early‐onset, severe obesity and clinical features of genetic obesity (e.g., hyperphagia, neurodevelopmental differences, dysmorphic features); however, only a small percentage of individuals who meet these criteria undergo testing, potentially owing to limited availability, overlapping symptoms with other obesity types, and infrequent use of genetic testing during diagnosis. Diagnosing hyperphagia may be challenging, as no guidelines have been established for individuals with MC4R pathway diseases. Identifying these individuals is crucial to addressing the challenges of hyperphagia and associated obesity, which often limit quality of life and place overwhelming burdens on patients and families.

**Study Importance**

**What is already known?**

- In 2014, Heymsfield et al. published a review on hyperphagia based on the proceedings of the 2nd International Conference on Hyperphagia [1].
- Most reviews on hyperphagia published to date focus on Prader‐Willi syndrome and do not explore the presentation or diagnosis of hyperphagia in other disease states.

**What does this review add?**

- There are a variety of diagnostic tools available for assessing overeating in the clinic, although few have been developed specifically for assessing hyperphagia in patients with obesity owing to rare melanocortin‐4 receptor pathway diseases.

**How might these results change the direction of research or the focus of clinical practice?**

- This review provides a guide for clinicians on available hyperphagia assessment tools and how to recognize and diagnose the associated symptoms.
- The authors present a call to action for expanded, validated hyperphagia assessment tools that are practical for use in the clinic, as well as the development of established standardized guidelines for diagnosis and treatment.

## INTRODUCTION

Obesity is a multifactorial disease with diverse etiologies, including social, environmental, behavioral, and genetic factors [2]. Monogenic and syndromic obesities with established etiologies are caused by variants in a small number of genes (as few as one gene) and/or deletions of chromosomal regions encompassing genes that are involved in key obesity pathogenesis pathways [3, 4]. Patients with these genetic variants or deletions generally exhibit early‐onset, severe obesity. The pathophysiology of obesity related to genetic dysfunction involves disruptions in key regulatory pathways of energy balance, which can be affected to varying degrees, depending on the specific genetic abnormality and its functional consequences [4].

Individuals with melanocortin‐4 receptor (MC4R) pathway diseases represent a distinct subset among those with monogenic and syndromic obesities [4, 5, 6]. A hallmark of such MC4R pathway diseases is the presence of hyperphagia, a pathologic condition characterized by heightened and prolonged hunger, longer time to reach satiation, shorter duration of satiety, severe preoccupation with food, abnormal food‐seeking behaviors, and distress and/or functional impairment when food is unavailable; these symptoms can be extreme and persistent [1, 2, 3, 5, 7, 8, 9, 10, 11, 12, 13, 14, 15].

In contrast to behavioral, psychological, and/or social challenges in other forms of overeating that are regulated by conscious or higher‐order brain functions, hyperphagia results from dysregulation of hypothalamic pathways involving energy balance [1, 16, 17, 18, 19]. This review focuses on the unmet need for fit‐for‐purpose tools to assess and diagnose hyperphagia in patients with monogenic, syndromic, and acquired forms of obesity associated with MC4R pathway diseases that affect energy balance.

## HYPERPHAGIA AND THE MC4R PATHWAY

### Central regulation of eating behavior

Eating behavior involves complex neuronal signaling networks located within and outside the hypothalamus that regulate food intake, reward pathways, energy expenditure, body weight, and glucose homeostasis [2, 17, 20]. The hypothalamic MC4R pathway has an essential role in the control of appetite/hunger and satiety signals (reviewed in depth in Yazdi et al. [2, 21]) [21]. This pathway comprises key molecules, receptors, and processing enzymes that are encoded by genes such as *GNAS*, *LEP*, *LEPR*, *MC4R*, *PCSK1*, *POMC*, *SH2B1*, and *SIM1*, as well as key Bardet‐Biedl syndrome (BBS)‐associated genes [2, 22, 23, 24]. Disruptions in MC4R signaling resulting from variants in these key genes can lead to hyperphagia [1, 2, 22, 24]. Although clinical presentations may vary among these MC4R pathway diseases, hyperphagia associated with obesity is the most consistent characteristic (Table 1) [1, 2, 3, 4, 10, 11, 12, 13, 14, 15, 23].

**TABLE 1:** Clinical characteristics of MC4R pathway diseases associated with hyperphagia and obesity.

Disease	Hyperphagia	Early‐onset obesity	Growth	Other possible clinical characteristics
Monogenic MC4R pathway diseases				
LEP deficiency [2, 25, 26]	✓	✓	Normal linear growth	Hypogonadotropic hypogonadism, hyperinsulinemia, altered immune function
LEPR deficiency [27, 28, 29]	✓	✓	Normal linear growth with reduced adult height	Hypogonadotropic hypogonadism, hypothyroidism, severe bacterial infections due to defective T‐cell immunity
MC4R deficiency [12, 30, 31]	✓	✓	Accelerated linear growth during childhood	Hyperinsulinemia, increased lean mass
PCSK1 deficiency [32, 33]	✓	✓	Failure to thrive in early infancy	Hypoglycemia, hypothyroidism, adrenocorticotropic hormone deficiency, diarrhea in early infancy
POMC deficiency [34, 35, 36]	✓	✓	Normal childhood height trajectory	Adrenocorticotropic hormone deficiency, mild hypothyroidism, red/orange hair, light skin
SRC1 deficiency^a^ [10]	✓	✓	No abnormalities reported	Impaired leptin‐induced POMC expression, polycystic ovarian syndrome, low testosterone and gonadotropin levels, previous fractures from minor incidents, liver fibrosis, diabetes/insulin resistance
SH2B1 deficiency [3, 11]	✓	✓	Reduced adult height	Hyperinsulinemia, delayed speech and language development
SIM1 deficiency [37, 38]	✓	✓	Short stature	Hypopituitarism, developmental delays, hypotonia, facial dysmorphisms
Syndromic MC4R pathway diseases				
AHO^b^ [23, 39]	✓	✓	Short stature	Round face, endocrine anomalies, brachydactyly
BBS [15, 40, 41]	✓	✓	Rapid weight gain in early childhood and sustained through adolescence	Hypogonadism, visual impairment, renal dysfunction, cognitive disabilities, polydactyly
PWS [42, 43]	✓	✓	Short stature	Neonatal hypotonia, developmental delays, hypogonadotropic hypogonadism
Acquired MC4R pathway diseases				
Hypothalamic obesity [44, 45, 46, 47]	✓		Linear growth may be reduced in early childhood	Daytime fatigue, decreased physical activity and energy expenditure, endocrine dysfunction, sleep disturbances

Abbreviations: AHO, Albright hereditary osteodystrophy; BBS, Bardet‐Biedl syndrome; LEP, leptin; LEPR, leptin receptor; MC4R, melanocortin‐4 receptor; PCSK1, proprotein convertase subtilisin/kexin type 1; POMC, proopiomelanocortin; PWS, Prader‐Willi syndrome; SH2B1, SH2B adaptor protein 1; SIM1, single‐minded homolog 1; SRC1, steroid receptor coactivator 1.

^a^ Associated with variants in *NCOA1*.

^b^ Associated with variants in *GNAS*.

### Differentiating hyperphagia from other overeating behaviors and disorders

There are many types of overeating behaviors and disorders (reviewed in depth in Heymsfield et al. and Haqq et al. [1, 16]), including occasional overeating, emotional overeating, hedonic overeating, binge eating, and hyperphagia [1, 7, 16, 18]. Occasional overeating/feasting is relatively common (e.g., a large holiday meal) [1]. Emotional overeating (i.e., overeating in response to stress or negative emotions) is often associated with overeating in response to external food‐related cues such as sight or smell during periods, including, but not limited to, stress, boredom, or anger [18, 39]. Taken together, these common forms of overeating are often termed “hedonic overeating.” Hedonic overeating is generally considered to be driven by opioid and dopamine signaling in reward areas such as the nucleus accumbens and is characterized by activity in reward pathways that override inhibitory and homeostatic signals to drive appetite despite satiety signaling occurring [17]. Disruption of the dopamine or opioid pathways that regulate neuronal systems associated with reward sensitivity, incentive motivation, conditioning, and self‐control results in eating past the point of satiation [20]. Binge eating is the consumption of a large amount of food with loss of control in the absence of hunger [19]. Binge eating is associated with marked distress and is characterized by recurrent episodes of excess food consumption (≥once per week for ≥3 months) [19]. In children, these overeating behaviors are rare, and it may be difficult to establish what constitutes excessive food consumption, but the presence of binge eating or loss of control of eating (without clearly excessive intake during episodes) is associated with excessive weight and/or fat mass gain [48]. Diagnostic criteria for binge eating disorder are defined in the *Diagnostic and Statistical Manual of Mental Disorders* (Fifth Edition) (DSM‐5) [19]; however, hyperphagia has no agreed‐upon criteria for diagnosis.

Hyperphagia is considered the rarest and most extreme form of overeating and may be associated with waking during the night to seek food, foraging through trash for food, stealing food, or eating nonfood items [1, 7, 8, 9, 16, 49]. Although excessive or uncontrolled eating is also observed in other overeating behaviors and disorders, hyperphagia differs in the frequency, duration, and severity of food‐seeking behaviors [1]. Hyperphagia is not usually accompanied by inappropriate compensatory behaviors (e.g., fasting, purging, excessive exercising) that may present in other overeating behaviors and disorders such as bulimia nervosa [19]. Hyperphagia severity itself exists on a spectrum within and across diseases; for example, patients with Prader‐Willi syndrome (PWS) often exhibit aggressive hyperphagic behaviors that can lead to life‐threatening complications that are not typically exhibited by patients with MC4R deficiency or BBS [15, 16]. The overall prevalence of hyperphagia within individual MC4R pathway diseases has not been well characterized, potentially because of heterogeneity in presentation, lack of awareness, and lack of standardized diagnosis and measurement tools. The persistent insatiable hunger and impaired satiety associated with hyperphagia are, at least in some cases, linked to an underlying disorder characterized by dysregulation of the MC4R pathway [2, 3, 4, 10, 11, 12, 13, 14, 15]. Critically, hyperphagia has profound negative impacts on health‐related quality of life and is often managed through strict environmental (i.e., food availability) regulation [1, 8, 9, 50, 51].

### Monogenic MC4R pathway diseases associated with hyperphagia and obesity

Monogenic obesity is defined as obesity that results from disruption in the function of a single gene [4]. Loss‐of‐function variants in genes in the leptin–melanocortin pathway, including *LEP*, *LEPR*, *POMC*, *PCSK1*, *NCOA1* (associated with steroid receptor coactivator 1 deficiency), *SH2B1*, and *SIM1*, may result in monogenic MC4R pathway diseases and cause hyperphagia and early‐onset obesity [2, 3, 4, 52].

In an epidemiologic analysis of known and predicted loss‐of‐function variants in *POMC*, *PCSK1*, and *LEPR*, the number of individuals in the United States who had MC4R pathway diseases associated with obesity was estimated to be 12,800 out of a total population of 300 million (0.004%), most of whom remain undiagnosed [21]. However, the true prevalence of MC4R pathway diseases is unknown because genetic testing is often unavailable or not obtained for individuals with obesity [21, 53]. The underutilization of genetic testing for patients with MC4R pathway diseases may result from a lack of understanding or awareness of these diseases among health care professionals [53]. Consequently, clinicians infrequently consider genetic testing as a diagnostic method and may misdiagnose patients as having obesity without a monogenic or oligogenic origin [53].

### Syndromic MC4R pathway diseases associated with hyperphagia and obesity

Syndromic obesity is severe obesity associated with additional phenotypes. PWS and BBS are the syndromes most frequently linked to obesity; however, >100 syndromic forms of obesity have been identified, including Albright hereditary osteodystrophy (AHO) [4, 23].

PWS is the most commonly recognized form of syndromic obesity, occurring with a prevalence of one in ten thousand to thirty thousand [1, 42]. PWS is characterized by neonatal hypotonia and feeding difficulties, failure to thrive, behavioral issues, developmental delays, endocrinopathies, predisposition to early‐onset obesity, and hyperphagia [42, 54]. PWS results from loss of multiple paternally expressed genes (maternal alleles are silenced or imprinted) in a critical region of chromosome 15q11‐13 and is most commonly due to a chromosomal deletion (70%–75%), maternal uniparental disomy (20%–30%), or imprinting defect (2%–5%) [43, 55]. Mice lacking *Magel2*, a gene encoded in chromosome 15q11‐13, have shown impaired leptin response and reduced leptin receptor (LEPR) cell‐surface trafficking in proopiomelanocortin (POMC)‐expressing neurons, suggesting potential implication of the MC4R pathway [55]. Additionally, the loss of the paternal *SNORD115* small nucleolar gene cluster in patients with PWS may alter the expression of serotonin receptor 2C and thereby dysregulate MC4R signaling, given that these serotonin receptors are hypothesized to regulate satiety and food intake through their activation in POMC neurons [1, 56, 57].

BBS is a genetic obesity syndrome in which the MC4R pathway is implicated in the pathogenesis of hyperphagia and obesity [4, 22, 24]. Prevalence of BBS is estimated to range from 1 in 100,000 to 1 in 160,000 in North America and Europe [58]. In BBS, variants in at least 1 of ≥26 characterized genes result in defective cilia function in particular cell types, including neurons and retina, which can lead to developmental delays, retinal degeneration, renal failure, and/or genital abnormalities [40, 59]. Variants in BBS‐associated genes can impair trafficking of membrane proteins, including those involved in appetite regulation (e.g., *LEPR*), and result in hyperleptinemia, consistent with leptin resistance and impaired signaling in the MC4R pathway [22, 24]. Altered leptin signaling can disrupt satiety and food intake, leading to hyperphagia and early‐onset obesity [22, 24].

AHO is another example of syndromic obesity and is associated with variants in *GNAS*, which encodes the stimulatory G‐protein α subunit of G‐protein–coupled receptors. Patients with AHO can be categorized based on whether they have hormone resistance, known as pseudohypoparathyroidism (i.e., variants on maternally inherited alleles), or no hormone resistance, known as pseudopseudohypoparathyroidism (i.e., variants on paternally inherited alleles) [23]. Children who inherit maternal, but not paternal, *GNAS* function‐altering variants frequently develop obesity. Among other effects, *GNAS* variants may impair signaling of MC4R, a G‐protein–coupled receptor, leading to hyperphagia and early‐onset obesity [23]. Patients with AHO can also present with developmental delays, brachydactyly, and short stature.

### Acquired MC4R pathway diseases associated with hyperphagia and obesity

Acquired hypothalamic obesity is characterized by rapid and excessive weight gain after hypothalamic injury [44, 45, 46, 47]. Damage to the hypothalamus can occur because of tumors or tumor therapy (i.e., surgery or radiotherapy) and can potentially affect the MC4R pathway [47]. This change in weight regulation may be caused by the accompanying symptoms that arise from damage to the hypothalamic nuclei, including daytime fatigue, decreased physical activity, decreased energy expenditure, hypothalamic–pituitary dysfunction leading to endocrine disorders, sleep disturbances, decreased satiety, and hyperphagia [44, 45, 46, 47].

## CLINICAL CHARACTERISTICS AND BURDEN OF HYPERPHAGIA IN MC4R PATHWAY DISEASES

Case reports and small case series of children with variants in *GNAS*, *LEP*, *LEPR*, *POMC*, *PCSK1*, or *MC4R* include descriptions of severe hyperphagia and constant, aggressive food‐seeking behaviors, including fighting with other children for food, lack of satiation, and impulsiveness around food [12, 23, 25, 27, 32, 34, 49, 60, 61]. Hyperphagia has been observed within the first few years of life in patients who have variants in *GNAS*, *LEP*, *LEPR*, *MC4R*, *NCOA1*, *PCSK1*, *POMC*, and *SH2B1* (including 16p11.2 deletions) [3, 10, 12, 25, 27, 28, 35, 49, 52, 61, 62].

Hyperphagic characteristics or symptoms are significantly greater in patients with BBS than in control patients with nonsyndromic obesity [15]. Such symptoms are observed early in life [9, 15]. In one survey, most caregivers of patients with BBS (91%; 10/11) reported observing hyperphagic characteristics in their patient before age 5 years [15]. In a separate survey, the CAREgiver Burden in BBS (CARE‐BBS) study, caregivers of patients with BBS reported first noticing symptoms of uncontrollable hunger at a median age of 8.7 years (*N* = 242) [9]. Patients with BBS have described their hyperphagia as constant, all‐consuming, and extreme, leading to an intense focus on food and persistent pursuit/consumption of food [8]. In the CARE‐BBS study, caregivers also reported observing hyperphagic behaviors throughout the day [9]. All hyperphagic behavioral measures were reported as occurring ≥1 time in the past 24 h in >80% of patients [9]. The most frequent behaviors were negotiating for food (90%) and waking for food during the night (88%) [9].

Hyperphagia has a broad negative effect on patients' quality of life and is considered one of the most distressing symptoms of MC4R pathway diseases [9, 41, 50, 51]. Hyperphagia can also cause distress and negatively affect the families and caregivers of patients with MC4R pathway‐associated obesity [1, 9, 41].

## DIAGNOSIS OF HYPERPHAGIA

### Suspecting hyperphagia associated with MC4R pathway diseases

Hyperphagia is a common characteristic across MC4R pathway diseases but may be underrecognized in a clinical setting because clinicians may concentrate on other differentiating phenotypes and may not recognize symptoms of hyperphagia [1, 3, 4, 10, 53, 62]. For example, patients with *MC4R* variants may have developmental or linear growth differences compared with patients with obesity who do not have MC4R deficiency [12, 30, 31, 52]. Patients with POMC deficiency can have fair skin, reddish hair, and cortisol deficiency, although these characteristics may be absent [34, 36]. Patients with *LEPR* variants may present with hypogonadism and failure to enter/complete puberty [27, 29]. Patients with homozygous or compound heterozygous *PCSK1* variants are characterized by infancy‐onset diarrhea, development of hypothalamic–pituitary dysfunction, and postprandial hypoglycemia caused by hyperproinsulinemia [32, 49]. Patients with *LEP* variants can present with hyperinsulinemia and history of infections, especially upper respiratory tract infections, potentially resulting from abnormal T‐cell counts and function [25, 26]. Along with focusing on these other clinical characteristics, clinicians treating patients suspected of having MC4R pathway diseases should also perform screening for hyperphagia.

Whereas the variants described are associated with severe, persistent hyperphagia, the presence of other, more subtle changes in these gene systems (e.g., epigenetic modification owing to alterations in methylation that affect expression) or variants with milder functional consequences might display obesity with less severe hyperphagia and/or additional phenotypes [63, 64]. Heterozygous variants are often associated with less severe phenotypes or symptoms compared with homozygous and compound heterozygous variants [12, 13, 14]. Furthermore, presence of multiple homozygous or compound heterozygous variants in the MC4R pathway genes *LEPR*, *POMC*, or *PCSK1* can have cumulative allelic burden effects on body weight [21].

### Available tools to diagnose overeating behaviors and disorders

#### Measures developed for MC4R pathway diseases

Accurate quantitative assessment of hyperphagia is challenging. Multiple instruments have been designed to assess overeating, but measures of hyperphagia are limited, with most of these tools developed in patients with PWS [7, 39, 54, 65, 66, 67, 68]. Available tools to measure overeating behaviors and disorders in patients with PWS include the Dykens' Hyperphagia Questionnaire; the Hyperphagia Questionnaire for Clinical Trials; 10‐cm–long visual analog scales to rate hunger, fullness, and desire to eat; the Food‐Related Problems Questionnaire; the Developmental Behavior Checklist‐Monitoring Version questionnaire; the Oxytocin Study Questionnaire; and the Pediatric‐Youth Hyperphagia Assessment for PWS questionnaire [7, 54, 66, 69, 70, 71, 72].

The Dykens' Hyperphagia Questionnaire, a 13‐item measure of food‐seeking behaviors, was developed to measure hyperphagic drive, behavior, and severity in pediatric and adult patients with PWS [7]. This questionnaire allows robust examination of the correlates and trajectories of hyperphagia and is completed by the parent or caregiver, as intellectual disability is a typical characteristic of PWS, and patients may be inconsistent or acquiescent in reporting their overeating behavior [7]. Responses for items assessing the frequency of hyperphagic behavior can range from “never” to “several times a day” [7]. A limitation of the Dykens' Hyperphagia Questionnaire is that medications, dietary regimens, and control of food environment are not considered [7].

The Hyperphagia Questionnaire for Clinical Trials is a nine‐item modified version of the Dykens' Hyperphagia Questionnaire for use in clinical trials of pediatric and adult patients with PWS [72]. It is also completed by caregivers or parents. Drawbacks of this questionnaire include restriction to observable behaviors that could change with treatment and assessment over a shorter period (i.e., during the previous 2 weeks) [72]. Additionally, patients may live in an environment where access to food is restricted or in which caregivers enforce strict food‐related routines such as preparing and supervising meals and snacks [73]. Therefore, certain hyperphagic behaviors such as waking up during the night to seek food, foraging through trash for food, or stealing food may not be apparent because patients lack these opportunities [73].

Visual analog scales, which are self‐reported, can be used to assess changes in hunger intensity before, during, and after food availability [7, 54, 74]. This method of assessment is limited by several factors. It is subjective in nature and restricted to patients who are not cognitively impaired, those who have undergone an intervention for hyperphagia, or those who have a prior report for comparison.

The Food‐Related Problems Questionnaire is a 20‐item questionnaire completed by parents or caregivers that assesses preoccupation with food, impairment of satiety, difficulty with self‐control, and other challenging behaviors of food‐related problems. The questionnaire was developed to compare adult patients with PWS with a control group of adult patients with intellectual disabilities. A limitation of this questionnaire is the requirement of a verbal response, including the option of “does not apply,” from respondents for some items, which could result in lower scores [66].

The Developmental Behavior Checklist‐Monitoring Version, a six‐item questionnaire completed by parents or caregivers to assess daily eating behaviors in pediatric and adult patients with PWS, assesses gorging of food, eating nonfood items, underreacting to pain, scratching/picking skin, obsessing about an idea or an activity, and temper outbursts [70]. The Oxytocin Study Questionnaire, completed by parents or caregivers, assesses changes in emotions, social and eating behaviors, and possible adverse effects in pediatric patients with PWS after oxytocin treatment [71]. The Pediatric‐Youth Hyperphagia Assessment for PWS is a parent‐reported 14‐item questionnaire to assess the verbal, behavioral, and social domains of pediatric patients with PWS over time; the scores for this assessment range from 14 to 66, with higher scores representing more severe hyperphagia [69].

Currently, only two measures have been developed and validated to diagnose hyperphagia in patients with an MC4R pathway disease outside of PWS. The recently developed Symptoms of Hyperphagia and Impacts of Hyperphagia questionnaires were created as part of the CARE‐BBS study to assess the emotional and physical burden of MC4R pathway‐related hyperphagia in patients with BBS and their caregivers [9, 75]. The Symptoms of Hyperphagia Questionnaire is a five‐item observer‐reported questionnaire to measure the frequency of hunger‐related behaviors observed by caregivers [9]. The Impacts of Hyperphagia Questionnaire is a 10‐item observer‐ and self‐reported questionnaire to measure the extent to which hunger behavior affects multiple aspects of life in patients and caregivers [9, 75]. The caregiver versions of both the Symptoms of Hyperphagia and Impacts of Hyperphagia questionnaires use proxy measures, which could influence results from the perspective of the caregiver [9]. Both questionnaires have been reported in an abstract to be psychometrically valid in caregivers of patients with BBS [76].

#### Measures developed for populations without MC4R pathway diseases

Available tools to measure overeating behaviors and disorders in populations without MC4R pathway diseases include the Three‐Factor Eating Questionnaire, the Power of Food Scale, the Dutch Eating Behavior Questionnaire, the Children's Eating Behavior Questionnaire (CEBQ), the Night Eating Questionnaire, the Yale Food Addiction Scale, and the Binge Eating Scale (Table 2) [39, 65, 67, 68, 77, 78]. These questionnaires were not developed or intended for use in those with neurocognitive deficits.

**TABLE 2:** Current eating behavior assessments.

Assessment	Development population	Additional MC4R pathway‐related study population(s)	Respondents	Description	Limitations
Dykens' Hyperphagia Questionnaire [7]	Pediatric and adult patients with PWS Age range: 4–51 y (mean [SD], 20.23 [11.3] y)	Pediatric patients with BBS [15] Pediatric patients with Alström syndrome [79] Pediatric patients with variants in *LEPR* or *MC4R* and 16.p11.2 deletions, including *SH2B1* [74]	Caregiver‐ or parent‐reported	13‐item questionnaire to assess food‐related preoccupations, including hyperphagic behavior, drive, and severity	Living situations (e.g., living with others in a more behavioral‐structured setting), medications, and dietary regimens are not considered
Hyperphagia Questionnaire for Clinical Trials [72, 73]	Pediatric and adult patients with PWS Age range: 12–65 y (mean [SD], 20.9 [7.8] y [72]); ≥5 y [73]	NA^a^	Caregiver‐ or parent‐reported	9‐item modified version of Hyperphagia Questionnaire for use in clinical trials to assess observable behavioral changes with treatment and over a shorter time period	Does not assess all aspects of hyperphagia such as food preferences or mealtime behaviors; may not be sensitive enough to detect changes in hyperphagia over time, especially in patients with mild‐to‐moderate hyperphagia; does not account for environmental factors such as food access or availability
10‐cm–long visual analog scale [54]	Adult patients with PWS Age range: 18–31 y (mean, 24 y)	NA^a^	Self‐reported	Questionnaire to assess rating changes in hunger intensity; desire to eat; and fullness before, during, and after food availability	Patients with intellectual impairment may have difficulty rating feelings
Food‐Related Problems Questionnaire [66]	Pediatric and adult patients with PWS Age range: 8–24 y	Pediatric and adult patients with SMS [80]	Caregiver‐ or parent‐reported	20‐item questionnaire to assess preoccupation with food, impairment of satiety, and other food‐related “challenging” behaviors in individuals with PWS	Verbal response, including option of “does not apply,” is required from respondents for some items, which could result in lower scores
Developmental Behavior Checklist‐Monitoring Version [70]	Pediatric and adult patients with PWS Age range: 12–29 y (mean [SD], 17.8 [4.77] y)	NA^a^	Parent‐reported	6‐item questionnaire to assess daily eating behaviors in patients with PWS undergoing treatment	Does not assess all aspects of behavioral problems, such as social skills or adaptive behavior
Oxytocin Study Questionnaire [71]	Pediatric patients with PWS Age range: 6–13.7 y (median, 9.3 y)	NA^a^	Parent‐reported	Assesses changes in emotions, social and eating behaviors, and possible side effects	Does not account for environmental factors such as food access or availability
Pediatric‐Youth Hyperphagia Assessment for PWS [69]	Pediatric patients with PWS Median (IQR) age: 10 (5–18) y	NA^a^	Parent‐reported	14‐item questionnaire to assess verbal, behavioral, and social domains of children and adolescents with PWS over time	Clinical stability was not confirmed, not suitable for evaluating hyperphagia in adults with PWS
Impacts of Hyperphagia^b^ Questionnaire [9, 75]	Pediatric and adult patients with BBS Age range: 2–30 y (mean [SD], 12 [3.7] y)	NA^a^	Caregiver‐ and patient‐reported	10‐item observer‐reported (5 items) and self‐reported (5 items) questionnaire to assess how hunger behavior affects multiple aspects of life in patients and caregivers	Use of proxy measures has potential to influence results from perspective of caregiver
Symptoms of Hyperphagia^b^ Questionnaire [9]	Pediatric and adult patients with BBS Age range: 2–30 y (mean [SD], 12 [3.7] y)	NA^a^	Caregiver‐reported	5‐item observer‐reported questionnaire to measure frequency of hunger‐related behavior observed by caregivers	Use of proxy measures has potential to influence results from perspective of caregiver
Three‐Factor Eating Questionnaire [67]	Adult participants whose eating was restrained (i.e., dieters) or unrestrained Age range: 17–77 y (mean [SD], 44 [12.8] y)	NA^a^	Self‐reported	51‐item questionnaire to detect individual and group differences of eating behavioral restraint (Factor I), liability of behavior and weight (Factor II), and perceived hunger and associated behavioral effects (Factor III)	When prior inhibition is not a prerequisite, Factor II requires continuous review for accurate interpretations regarding behavioral and weight lability
Power of Food Scale [65, 81]	Adult participants with healthy weight, overweight, or obesity Age range: 18–42 y [65] (mean [SD], 46.3 [11.0] y)	NA^a^	Self‐reported	21‐item questionnaire to assess psychological effect of food proximity in environments where food is abundant, including responsiveness to food that is not present but available, food that is present but has not been tasted, and food after tasting but not consuming	Cannot be used for patients in environments where food availability is considerably different
Dutch Eating Behavior Questionnaire [39]	Adult participants with and without obesity Mean (SD) age: men, 30.8 (5.2) y; women, 31.1 (8.4) y	NA^a^	Self‐reported	33‐item questionnaire to assess restrained, emotional, and external eating	Patients with obesity may experience social pressure to lose weight, causing higher restrained eating scores and lower emotional/external eating scores
CEBQ [68]	Pediatric participants Age range: 2–7 y (mean [SD], 4.2 [1.3] y)	NA^a^	Parent‐reported	35‐item questionnaire to assess parent‐reported eating style in children, including response to food/drinks, food enjoyment, satiety, slowness, fussiness, and emotional overeating/undereating	Scales had internal consistency and reliability
Night Eating Questionnaire [77, 82]	Adult participants with obesity Mean (SD) age: 44 (12) y [77] Mean (SEM) age: student group, 18.7 (0.1) y; community group, 42.9 (0.6) y [82]	NA^a^	Self‐reported	18‐item questionnaire to assess behavioral and psychological symptoms of night eating syndrome	Does not assess all aspects of night eating syndrome, such as distress associated with night eating
Yale Food Addiction Scale [78, 83]	Adult participants Mean (SD) age: 20.11 (1.38) y [78]	NA^a^	Self‐reported	Assesses eating behaviors aligned to those seen in DSM‐5 diagnostic criteria of addiction for alcohol and substance dependence	Based on diagnostic criteria for substance dependence, objectively measuring food addiction is difficult
Binge Eating Scale [84]	Adult participants with obesity Mean (SD) age: 41.9 (11.3) y	NA^a^	Self‐reported	Assesses binge eating severity	Nonspecified time frame of items, difficulty identifying individuals without binge eating disorder and the frequency of binge eating

Abbreviations: BBS, Bardet‐Biedl syndrome; CEBQ, Children's Eating Behavior Questionnaire; DSM‐5, *Diagnostic and Statistical Manual of Mental Disorders* (Fifth Edition); MC4R, melanocortin‐4 receptor; NA, not applicable; PWS, Prader‐Willi syndrome; SMS, Smith‐Magenis syndrome.

^a^ The systematic literature review did not identify the use of the assessment in additional MC4R pathway‐related study populations.

^b^ The Impacts of Hyperphagia and Symptoms of Hyperphagia questionnaires are in development to address the need for proper, validated assessments to evaluate hyperphagia. Note that assessments may be used primarily in a research setting.

The Three‐Factor Eating Questionnaire evaluates cognitive dietary restraint, disinhibition, and hunger‐related eating [67]. The 51‐item self‐reported questionnaire was developed for adult individuals whose eating was restrained (i.e., dieters) or unrestrained and detects individual and group differences of eating and associated behavioral effects according to three measures: behavioral restraint (Factor I), disinhibition (Factor II), and perceived hunger (Factor III) [67]. The Child Three‐Factor Eating Questionnaire was subsequently developed and validated to evaluate similar parameters (i.e., uncontrolled eating, emotional eating, and cognitive restraint) in children and adolescents [85].

The Power of Food Scale measures appetite for, rather than consumption of, palatable foods and may be useful for measuring the hedonic effect of highly palatable food environments [65]. This 21‐item self‐reported instrument was validated in an adult population in which the majority (79.5%) had a body mass index (BMI) < 25 kg/m^2^ and was later tested in adult participants with obesity [65, 81]. The questionnaire assesses the psychological effect of food proximity in environments where food is abundant, including responsiveness to food that is not present but available, food that is present but has not been tasted, and food after tasting but not consuming [81]. A shortcoming of the scale is that it cannot be used to compare participants in environments where food availability differs considerably (i.e., participants must be living in environments where food abundance is the same or similar) [81].

The Dutch Eating Behavior Questionnaire is a 33‐item self‐report questionnaire that measures dietary restraint, emotional eating, and external eating behaviors (i.e., influence of external food properties such as taste and smell) [39]. It was developed for adults with normal weight or obesity [39]. Individuals with overweight or obesity may experience social pressure to lose weight, which can cause higher restrained eating scores and thus should be acknowledged as a limitation of this questionnaire [39].

The CEBQ is a parent‐reported 35‐item questionnaire that assesses eating style in children, including response to food/drinks, food enjoyment, satiety, slowness, fussiness, and emotional overeating/undereating [68]. When assessed in 208 children aged ≤9 years, CEBQ scales had internal consistency and reliability, except for emotional overeating/undereating; however, predictive value was not established [68]. In another study of 555 children aged 2 to 6 years, CEBQ items related to food responsiveness predicted a higher BMI *z* score after 1 year, and a combination of all CEBQ items proved to have clinically meaningful BMI predictions [86]. These findings emphasize the need to interpret the correlations among factors carefully when using multiple regression models for clinically relevant predictions with the CEBQ [86].

The Night Eating Questionnaire is an 18‐item self‐report questionnaire to assess behavioral and psychological symptoms in adults with night eating syndrome [77]. In a study using this assessment, 46% reported an urge to eat between evening meal and sleep, 18% experienced hyperphagia during the evening, 11% reported eating during the night, and 18% felt distressed by their night eating [82].

The Yale Food Addiction Scale, which is self‐reported, assesses eating behaviors aligned to those seen in the DSM‐5 diagnostic criteria of addiction for alcohol and substance dependence [78]. The prevalence rates of food addiction, as determined by the Yale Food Addiction Scale, vary between ~5% and 10% in student and community samples and are 7% in children. However, food addiction may be overestimated as a result of potential selection bias, especially in website‐based studies, and these estimates should be interpreted with caution [83].

The Binge Eating Scale is a self‐reported measure to assess binge eating severity in adults with obesity [84]. The Binge Eating Scale is accurate for initial screening and diagnosing binge eating disorder and has demonstrated test–retest reliability, but it is not recommended for use as the only measure for diagnosis because it has a high rate of misdiagnosis [84]. Limitations of the measure include a nonspecified time frame of questionnaire items, difficulty identifying individuals without binge eating disorder, and lack of questions about identifying binge eating frequency [84]. The Adolescent Binge Eating Scale has also been used to identify individuals aged 12 to 18 years with obesity who are at high risk of binge eating disorder incidence [87].

Finally, the relative reinforcing value has been assessed for ad libitum eating tasks and can be interpreted to estimate how much food consumption contributes to the reinforcement of individual food behaviors [88].

## HYPERPHAGIA DIAGNOSIS CHALLENGES

### Challenges with clinical recognition of hyperphagia

Given the lack of a universally accepted hyperphagia definition or measure for diagnosing it in patients with MC4R pathway diseases, a standardized definition that can distinguish hyperphagia from other overeating behaviors and disorders is needed to improve diagnosis [1]. Hyperphagia has been identified as an early characteristic and hallmark of MC4R pathway diseases [6, 9]. Because of the rarity of MC4R pathway diseases, hyperphagia may be misdiagnosed as a more common or well‐known overeating behavior or disorder or overlooked owing to other health problems such as obesity‐related comorbidities [1]. Hyperphagia may additionally be mistaken for food choice impulsivity, which is associated with a tendency to react impulsively during heightened emotional states and delayed discounting through devaluation of future rewards [89, 90]. Hyperphagia may also be underrecognized or appear to decrease in patients who are in highly restrictive food environments (e.g., a lock on the refrigerator) [60]. Furthermore, patients with these diseases carry the genetic variants from birth and may not recognize their symptoms as abnormal; therefore, they may not report them to a physician [8]. Genetic testing, timely recognition, and diagnosis are critical to initiate earlier and appropriate, individualized management of hyperphagia [1, 6, 9].

### Challenges within MC4R pathway disease patient populations

Available tools to assess and diagnose overeating behaviors or disorders and dysregulated eating have traditionally not been fit for purpose or intended for use in patients with MC4R pathway diseases and thereby may measure symptoms and behaviors that are not seen in patients with such diseases [39, 67, 68, 78, 81]. Furthermore, hyperphagic symptoms and behaviors may go unrecognized owing to the rarity of MC4R pathway diseases [1]. In cultures in which overeating is customary or encouraged, patients may not recognize abnormal overeating behaviors [91, 92]. Similarly, in a single‐child household, parents may not recognize abnormal overeating behaviors as unusual given their lack of comparative experience.

Assessments that use proxy‐ or caregiver‐reported measures may be influenced from the perspective of the respondent [9, 75]. However, patients with MC4R pathway diseases will often present with intellectual disabilities, which may necessitate a proxy‐ or caregiver‐reported assessment [9, 40]. For young children with hyperphagia, proxy‐ or caregiver‐reported objective measures may be just as accurate as if the patients responded for themselves [9]. Assessing hyperphagia in adults can be difficult because they may exhibit less overt and less frequent behavioral symptoms compared with children [93, 94]. Adults may also demonstrate greater self‐restraint, which could make food‐seeking actions such as stealing food less noticeable. Volunteer‐based surveys may also have biases. Individuals who volunteer to take part in surveys may have more awareness of their own or their patients' hyperphagia‐associated disease and therefore may be more motivated to participate [9].

### Challenges with subjectivity of hunger

The nature of rating hunger itself is subjective [50]. Furthermore, responses can vary across different assessments and within an assessment of the same individual over the course of their disease [50]. Such variability occurs because of patients' ability to adapt to their condition (i.e., response shifting) and can also contribute to a baseline that is different from that of the general population [50]. The underlying cause of hyperphagia in genetic MC4R pathway diseases is congenital [2]; therefore, patients may be unaware that their severe insatiable hunger is abnormal until after receiving effective hyperphagia treatment [8]. This elevated baseline hunger compared with that of individuals without MC4R pathway variants may also make the quantification of hunger difficult [15, 66].

## FUTURE DIRECTIONS TO IMPROVE HYPERPHAGIA DIAGNOSIS

### Need for clinician education

Because MC4R pathway diseases are rare, many health care professionals may not be well versed or aware of the role that genetics plays in hyperphagia and obesity [5]. Increased education for clinicians and enhanced awareness of hyperphagic clinical features may improve the chance that a patient will be referred for genetic testing and diagnosis [95]. Management of hyperphagia‐associated obesity could lead to improved long‐term health outcomes and quality of life (e.g., sleep, relationships with family, school/work performance, leisure activities), as weight loss in patients with overweight or obesity reduces risk factors of cardiometabolic disease [9, 50, 51, 96].

Understanding the mechanisms of hunger and satiety can help individuals with MC4R pathway diseases and their caregivers by enabling them to improve management of their health (e.g., endocrine disorders, weight loss), shifting the clinical focus toward addressing their hunger (e.g., hyperphagic drive) and empowering individuals to improve their affect through the recognition that their condition originates from a biological basis [5, 6, 9, 50, 95].

### Need for consensus guidelines

There are no consensus guidelines for diagnosis of hyperphagia in patients with MC4R pathway diseases [1]. The Endocrine Society recommends genetic testing for individuals with early‐onset, severe obesity (aged ≤5 years) who have clinical features such as extreme hyperphagia and/or family history of severe obesity [6]. However, one study found that only 8% of patients seen in obesity clinics who met the criteria for genetic screening had undergone such testing [97]. Failure to test may be attributable to cost barriers for families, lack of genetic testing referrals, lack of awareness of underlying disease state or hyperphagia, and the overlap of clinical symptoms that can be mistaken for other forms of obesity [5, 53]. Improving testing rates may require that practitioners learn how to evaluate for genetic causes of obesity; a diagnosis of hyperphagia may help identify individuals with underlying genetic causes of obesity [6]. Health care professionals should assess patients for hyperphagia by identifying aberrant food‐seeking behaviors, including insatiable hunger, impaired satiety, preoccupation with food, or increased food intake [5].

### Novel tools for assessing hyperphagia in MC4R pathway diseases

As described earlier, the Symptoms of Hyperphagia and Impacts of Hyperphagia questionnaires were shown to be psychometrically validated in caregivers of patients with BBS, an MC4R pathway disease; therefore, these tools may be clinically useful to assess other MC4R pathway diseases [9, 75, 76]. Additional research is needed across settings and in other MC4R pathway diseases to further confirm the psychometric properties, including responsiveness and sensitivity to change over time [76].

## METHODS

**FIGURE 1:**
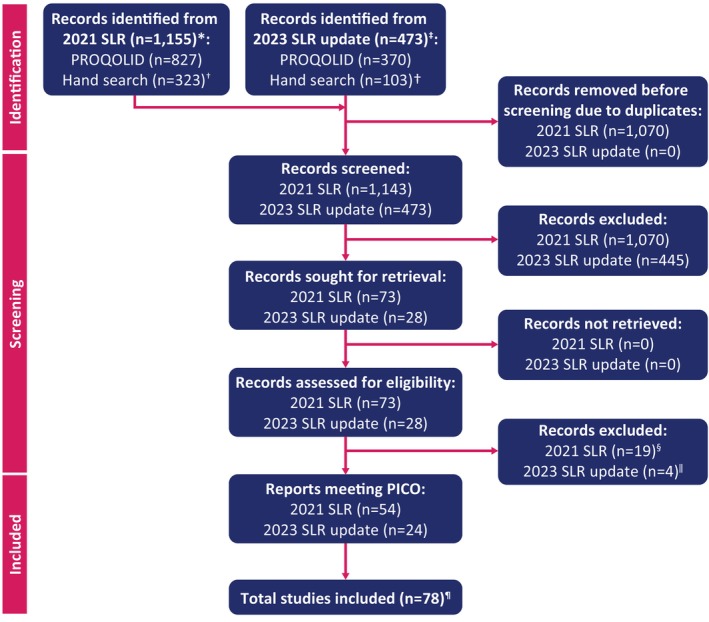
Identification of studies via databases, registers, and other methods. *Searches were conducted on September 29, 2021. ^†^Includes trial registries, citation checking of identified SLRs, and relevant conferences, as described in *Methods*. ^‡^Searches were conducted on August 7, 2023. ^§^Records were excluded based on outcome (*n* = 5) or due to duplicates (*n* = 14). ^‖^Records were excluded based on outcome (*n* = 3) or due to duplicates (*n* = 1). ^¶^See online Supporting Information for the full list of studies included in the SLR. PICO, patients, interventions/comparators, outcome; PROQOLID, Patient‐Reported Outcome and Quality of Life Instruments Database; SLR, systematic literature review. [Color figure can be viewed at wileyonlinelibrary.com]

Publications summarized in this narrative review were identified by literature searches and the authors. A systematic literature review was conducted to identify existing hyperphagia assessments in published literature, clinical trials, and conference abstracts to describe the existing approaches to quantify and assess hyperphagia (Figure 1). Patients, interventions/comparators, and outcome (PICO) elements were used to select relevant studies (online Supporting Information). Search terms, informed by the PICO elements, included both Medical Subject Headings and free‐text terms used to search the title, abstract, and author keywords to capture the highest proportion of relevant articles. The search strategy was developed in PubMed and translated to the Patient‐Reported Outcome and Quality of Life Instruments Database and ClinicalTrials.gov database. Citation searching and key author searching were used in addition to key conference searches. Established international guidelines for conducting systematic reviews guided the systematic literature review [98, 99].

## CONCLUSION

Dysfunction of the MC4R pathway results in hyperphagia and reduced energy expenditure, contributing to early‐onset, severe obesity. The associated hyperphagia is a pathophysiological driver of severe obesity in patients with MC4R pathway diseases. It has significant negative effects on health and quality of life for patients and their families. There is a need to understand when and how a patient with obesity should be assessed for hyperphagia. Although clinical assessments for hyperphagia are available, they are limited, and validated tools for such evaluation need to be developed and implemented, including across a spectrum of developmental ages. A better understanding of the features of genetic diseases can help identify and diagnose an individual's MC4R pathway disease and hyperphagia. Recommended guidelines and appropriate diagnostic tools should be adapted, especially as our understanding and continued advances in genetics identify novel genes that are associated with early‐onset, severe obesity.

## AUTHOR CONTRIBUTIONS

M. Jennifer Abuzzahab, Beatrice Dubern, Anthony P. Goldstone, Andrea M. Haqq, Steven B. Heymsfield, Jennifer L. Miller, Jesse Richards, Martin Wabitsch, and Jack A. Yanovski contributed to the conceptualization and design of the study. M. Jennifer Abuzzahab, Anthony P. Goldstone, Andrea M. Haqq, Jennifer L. Miller, Jesse Richards, and Jack A. Yanovski contributed to the analysis and interpretation of the results. All authors contributed to the writing of the drafts and provided final approval of the manuscript.

## CONFLICT OF INTEREST STATEMENT

M. Jennifer Abuzzahab's institution has received research support from Ascendis Pharma; Endo Pharmaceuticals Inc., Lumos Pharma, Novo Nordisk A/S, Pfizer Inc., Rhythm Pharmaceuticals, Inc., and Soleno Therapeutics, Inc., and she has received compensation for consulting, advisory boards, and speaking or educational engagements from Ascendis Pharma, Endo Pharmaceuticals Inc., Pfizer Inc., and Rhythm Pharmaceuticals, Inc. Beatrice Dubern is a consultant for Novo Nordisk A/S and primary investigator for Rhythm Pharmaceuticals, Inc. Anthony P. Goldstone has been a consultant for Evidera, Inc., Helsinn Healthcare SA, Idera Pharmaceuticals, Rhythm Pharmaceuticals, Inc., Soleno Therapeutics, Inc., Tonix Pharmaceuticals, and Veda Ventures; has been an advisory board member for Millendo Therapeutics and Radius Health; has been a member of the Data Safety Monitoring Committee for Novo Nordisk A/S; has received speaker honoraria from Novo Nordisk A/S and Rhythm Pharmaceuticals, Inc.; and has been a Principal Investigator for clinical trials sponsored by Millendo Therapeutics, Rhythm Pharmaceuticals, Inc., and Soleno Therapeutics, Inc. Andrea M. Haqq has received grants from the Weston Family Microbiome Initiative; is an advisory board member for the Foundation for Prader‐Willi Research, the 2023 Novo Nordisk Pediatric Expert Obesity National, and Rhythm Pharmaceuticals, Inc.; and is a trial investigator for Acadia Pharmaceuticals Inc., Eli Lilly and Company, and Rhythm Pharmaceuticals, Inc. Jesse Richards serves on speaker bureaus for Eli Lilly and Company, Novo Nordisk A/S, and Rhythm Pharmaceuticals, Inc. and is an independent consultant for Palatin Technologies, Inc. Steven B. Heymsfield is a medical advisory board member for Tanita Corp., Amgen plc, Novo Nordisk A/S, Versanis Bio, and Medifast and has served as an Amazon Scholar. Jennifer L. Miller has received study funding from Rhythm Pharmaceuticals, Inc., Soleno Therapeutics, Inc., and Harmony Biosciences. Martin Wabitsch has served as a consultant and speaker for Rhythm Pharmaceuticals, Inc. Jack A. Yanovski reports grant support from Soleno Therapeutics, Inc., and Rhythm Pharmaceuticals, Inc., for obesity‐related projects, as well as material support for research from Hikma Pharmaceuticals plc and Versanis Bio.

## Supporting information


**DATA S1:** Supplementary Information.

## Data Availability

Data sharing is not applicable to this article as no new data were created or analyzed in this study.
