# Supplementary material for: Improving the diagnosis of hyperphagia in melanocortin‐4 receptor pathway diseases
Source: Obesity (Silver Spring). 2025 Jun 17;33(7):1217–31. doi: 10.1002/oby.24287 (PMC12210103; doi:10.1002/oby.24287)
Supplement: Supplementary file 1 — DATA S1: Supplementary Information. [file OBY-33-1217-s001.docx]

**SUPPLEMENTAL MATERIAL**

**Improving the Diagnosis of Hyperphagia in Melanocortin-4 Receptor Pathway Diseases**

M. Jennifer Abuzzahab, MD^1^; Beatrice Dubern, PhD^2,3^; Anthony P. Goldstone, MD, PhD^4,5^;

Andrea M. Haqq, MD^6^; Steven B. Heymsfield, MD^7^; Jennifer L. Miller, MD^8^; Jesse Richards, DO^9^; Martin Wabitsch, MD^10^; Jack A. Yanovski, MD, PhD^11^

^1^Diabetes and Endocrine Center, Children’s Minnesota, St Paul, Minnesota, USA; ^2^Sorbonne Université, Trousseau Hospital, Assistance Publique-Hôpitaux de Paris, Paris, France; ^3^Sorbonne Université, Inserm, Nutrition and Obesities, Systemic Approaches Research Group, NutriOmics, Paris, France; ^4^PsychoNeuroEndocrinology Research Group, Division of Psychiatry, Department of Brain Sciences, Faculty of Medicine, Imperial College London, Hammersmith Hospital, London, UK; ^5^Imperial Centre for Endocrinology, Imperial College Healthcare NHS Trust, Hammersmith Hospital, London, UK; ^6^Division of Pediatric Endocrinology, University of Alberta, Edmonton, AB, Canada; ^7^Pennington Biomedical Research Center, Louisiana State University System, Baton Rouge, Louisiana, USA; ^8^Department of Pediatrics, University of Florida College of Medicine, Gainesville, Florida, USA; ^9^Department of Internal Medicine, University of Oklahoma at Tulsa, Tulsa, Oklahoma, USA; ^10^Division of Pediatric Endocrinology and Diabetes, Center for Rare Endocrine Diseases, Department of Pediatrics and Adolescent Medicine, University of Ulm, Ulm, Germany; ^11^Section on Growth and Obesity, Division of Intramural Research, *Eunice Kennedy Shriver* National Institute of Child Health and Human Development, National Institutes of Health, Bethesda, Maryland, USA

| **Table S1.** PICO Elements of Hyperphagia Assessment SLR | |
| --- | --- |
| **Element** | **Focus** |
| Patients | Experiencing hyperphagia under any indication* |
| Intervention | Any^†^ |
| Comparators | Any or none |
| Outcomes | Patient-reported outcomes of hyperphagia |
| Study design | Any^‡^ |
| Time frame | Any |
| Geography | No limit |
| Language | English-language abstract |
| Databases | PROQOLID and ClinicalTrials.gov |
| Other | Searches of the following conferences for the periods 2019-2021 (2021 SLR) and 2021-2023 (SLR update):   - Endocrine Society Annual Meeting - European Society for Paediatric Endocrinology Annual Meeting - American College of Neuropsychopharmacology - American Society for Metabolic and Bariatric Surgery Annual Meeting - The Obesity Society ObesityWeek - Obesity Medicine Association Conference - American Association of Clinical Endocrinology - 2nd International Conference on Hyperphagia - International Prader-Willi Syndrome Clinical Trial Consortium - Checking reference lists of relevant SLRs published in last 5 years - Checking reference lists of included studies |
| PICO, patients, interventions/comparators, outcome; PROQOLID, Patient-Reported Outcome and Quality of Life Instruments Database; SLR, systematic literature review. *Studies reporting assessments of eating behavior or hunger were also included if hyperphagia was mentioned as a measured feature. ^†^Include pharmacologic treatment as neoadjuvant/adjuvant/concurrent with other therapies. ^‡^SLRs were only used to check references. Non-SLRs were excluded. | |

**Studies Identified in the Systematic Literature Review**

Alaimo JT, Barton LV, Mullegama SV, Wills RD, Foster RH, Elsea SH. Individuals with Smith-Magenis syndrome display profound neurodevelopmental behavioral deficiencies and exhibit food-related behaviors equivalent to Prader-Willi syndrome. *Res. Dev. Disabil*. 2015;47:27-38.

Allas S, Caixàs A, Poitou C, Coupaye M, Thuilleaux D, Lorenzini F, Diene G, Crinò A, Illouz F, Grugni G, Potvin D, Bocchini S, Delale T, Abribat T, Tauber M. AZP-531, an unacylated ghrelin analog, improves food-related behavior in patients with Prader-Willi syndrome: a randomized placebo-controlled trial. *PLoS One*. 2018;13(1): e0190849.

Allas S, Mohideen P, Delale T, Lin V, Tremel N, Yeh M, Tauber M. SAT-100 trial-in-progress: ZEPHYR, a pivotal phase 2b/3 randomized, placebo-controlled study of livoletide, a novel unacylated ghrelin analog, for the treatment of hyperphagia and food-related behaviors in patients with Prader-Willi Syndrome. *J Endocr Soc*. 2019; 3(Supplement_1): SAT-100.

Argente J, van den Akker ELT, Ben-Ami M, Chung W, Goldstone A, McCormack S, Oral E, Pinhas-Hamiel O, Ohayon O, Scimia C, Stewart M, Yuan G, Wabitsch M, Farooqi S. Effects of setmelanotide on obesity, hunger, and safety in SH2B1 deficiency: a phase 2 trial. *Obesity*. 2021;29(S2):47-197.

Argente J, van den Akker E, Ben-Ami M, Chung WK, Goldstone AP, McCormack S, Oral EA, Pinhas-Hamiel O, Ohayon O, Scimia C. Effects of setmelanotide on obesity, hunger, and safety in SH2B1 deficiency: a phase 2 trial. Poster presented at: Obesity Week; November 1-5, 2021; Virtual.

Bouchard L, Drapeau V, Provencher V, Lemieux S, Chagnon Y, Rice T, Rao DC, Vohl MC, Tremblay A, Bouchard C, Pérusse L. Neuromedin beta: a strong candidate gene linking eating behaviors and susceptibility to obesity. *Am. J. Clin Nutr*. 2004;80(6):1478-1486.

Boutin P, Dina C, Vasseur F, Dubois S, Corset L, Séron K, Bekris LM, Cabellon J, Neve B, Vasseur-Delannoy V, Chikri M, Charles MA, Clément K, Lernmark Å, Froguel P. GAD2 on chromosome 10p12 is a candidate gene for human obesity. *PLoS Biology*. 2003;1(3):E68.

Brancati GE, Barbuti M, Calderone A, Fierabracci P, Salvetti G, Weiss F, Santini F, Perugi G. Prevalence and psychiatric comorbidities of night-eating behavior in obese bariatric patients: preliminary evidence for a connection between night-eating and bipolar spectrum disorders. *Eat. Weight Disord*. 2022;27(5):1695-1704.

Bravo GL, Poje AB, Perissinotti I, Marcondes BF, Villamar MF, Manzardo AM, Luque L, LePage JF, Stafford D, Fregni F, Butler MG. Transcranial direct current stimulation reduces food-craving and measures of hyperphagia behavior in participants with Prader-Willi syndrome. *Am. J. Med. Genet. B Neuropsychiatr. Genet*. 2016;171B(2): 266-275.

Çabal-Berthoumieu S, Molinas C, Liz Pacorina-Alfaro D, Bengvenul G, Glattard M, Faye S, Marion V, Diene W, Tauber M. Deprivation and obesity in patients with PWS. Poster presented at: 10th International Prader-Willi Syndrome Organisation Conference; November 13-17, 2019; Havana, Cuba.

Calugi S, Dalle Grave R, Marchesini G. Night eating syndrome in class II–III obesity: metabolic and psychopathological features. *Int. J. Obes*. *(Lond)*. 2009;33(8):899-904.

Cerú-Björk C, Andersson I, Rössner S. Night eating and nocturnal eating—two different or similar syndromes among obese patients? *Int. J. Obes. Relat. Metab. Disord*. 2001;25(3):365-372.

Cho SY, Kang D, Im M, Yang A, Kim MS, Kim J, Noh ES, Kwon EK, Choi E, Han S, Park YA, Kwak MJ, Kim Y, Cho J, Jin DK. Development and validation of the Pediatric-Youth Hyperphagia Assessment for Prader-Willi syndrome. *Epidemiol. Health*. 2022;44:e2022014.

Choquette AC, Lemieux S, Tremblay A, Drapeau V, Bouchard C, Vohl MC, Pérusse L. GAD2 gene sequence variations are associated with eating behaviors and weight gain in women from the Quebec family study. *Physiol. Behav.* 2009;98(4):505-510.

Clément K, van den Akker E, Argente J, Bahm A, Chung WK, Connors H, De Waele K, Farooqi IS, Gonneau-Lejeune J, Gordon G, Kohlsdorf K, Poitou C, Puder L, Swain J, Stewart M, Yuan G, Wabitsch M, Kühnen P. Efficacy and safety of setmelanotide, an MC4R agonist, in individuals with severe obesity due to LEPR or POMC deficiency: single-arm, open-label, multicentre, phase 3 trials. *Lancet Diabetes Endocrinol*. 2020;8(12):960-970.

Clement K, Argente J, Bahm A, Connors H, De Waele K, Gordon G, Puder L, Martos-Moreno G, Swain J, Farooqi S. Long-term weight and hunger reduction with setmelanotide in individuals with POMC deficiency. Poster presented at: Obesity Week; November 2-6, 2020; Virtual.

Consoli A, Çabal Berthoumieu S, Raffin M, Thuilleaux D, Poitou C, Coupaye M, Pinto G, Lebbah S, Zahr N, Tauber M, Cohen D, Bonnot O. Effect of topiramate on eating behaviours in Prader-Willi syndrome: TOPRADER double-blind randomised placebo-controlled study. *Transl. Psychiatry*. 2019;9(1):274.

Diene G, Angulo M, Hale PM, Jepsen CH, Hofman PL, Hokken-Koelega A, Ramesh C, Turan S, Tauber M. Liraglutide for weight management in children and adolescents with Prader-Willi syndrome and obesity. *J. Clin. Endocrinol. Metab*. 2022;108(1):4-12.

Dotson CD, Shaw HL, Mitchell BD, Munger SD, Steinle NI. Variation in the gene TAS2R38 is associated with the eating behavior disinhibition in Old Order Amish women. *Appetite*. 2010;54(1):93-99.

Dykens EM, Miller J, Angulo M, Roof E, Reidy M, Hatoum HT, Willey R, Bolton G, Korner P. Intranasal carbetocin reduces hyperphagia in individuals with Prader-Willi syndrome. *JCI Insight*. 2018;3(12):e98333.

Echeverri B, Kozak AT, Gildner DJ, Pickett SM. Night eating syndrome subtypes: differences in binge eating and food addiction symptoms. *Eat. Weight Disord*. 2023;28(1):3.

Einfeld SL, Smith E, McGregor IS, Steinbeck K, Taffe J, Rice LJ, Horstead SK, Rogers N, Hodge MA, Guastella AJ. A double-blind randomized controlled trial of oxytocin nasal spray in Prader Willi syndrome. *Am. J. Med. Genet. A*. 2014;164A(9): 2232-2239.

Ervin C, Norcross L, Mallya UG, Fehnel S, Mittleman RS, Webster M, Haqq AM, Haws RM. Interview-based patient- and caregiver-reported experiences of hunger and improved quality of life with setmelanotide treatment in Bardet-Biedl syndrome. *Adv. Ther*. 2023;40(5):2394-2411.

Farooqi S, Ben-Ami M, Kostopoulou E, Martos-Moreno GÁ, McCormack S, Oral EA, Pinhas-Hamiel O, Spiliotis BE, Ohayon O, Scimia C. Effects of setmelanotide on obesity, hunger, and safety in SRC1 insufficiency: a Phase 2 trial. Poster presented at: ObesityWeek^®^; November 1-5, 2021; Virtual.

Farooqi S, Ben-Ami M, Kostopoulou E, Martos-Moreno G, McCormack S, Oral E, Pinhas-Hamiel O, Spiliotis B, Ohayon O, Scimia C, Yuan G, Stewart M, Argente J. Effects of setmelanotide on obesity, hunger, and safety in SRC1 insufficiency: a phase 2 trial. [abstract]. *Obesity*. 2021;29(S2): 47-197.

Forsythe E, Haws R, Argente J, Beales P, Chirila C, Clément K, Dollfus H, Martos-Moreno GÁ, Gnanasakthy A, Buckley BC. Quality of life in patients with Bardet-Biedl Syndrome in a setmelanotide phase 3 trial. Poster presented at: Obesity Week; November 1-5, 2021; Virtual.

Forsythe E, Haws RM, Argente J, Beales P, Martos-Moreno GÁ, Dollfus H, Chirila C, Gnanasakthy A, Buckley BC, Mallya UG, Clément K, Haqq AM. Quality of life improvements following one year of setmelanotide in children and adult patients with Bardet–Biedl syndrome: phase 3 trial results. *Orphanet J. Rare Dis*. 2023;18(1):12.

Forsythe E, Mallya UG, Yang M, Huber C, Cala ML, Greatsinger A, Hagopian E, Pomeroy J, Haqq AM. Burden of hyperphagia and obesity in Bardet–Biedl syndrome: a multicountry survey. *Orphanet J. Rare Dis*. 2023;18(1):182.

Forsythe E, Mallya UG, Yang M, Huber C, Cala ML, Greatsinger A, Hagopian E, Pomeroy J, Haqq AM. Caregiver burden in Bardet-Biedl syndrome: findings from the CARE-BBS study. *Orphanet J. Rare Dis*. 2023;18(1):181.

Forsythe E, Mallya UG, Yang M, Huber C, Cala ML, Greatsinger A, Pomeroy J, Haqq AM. Caregiver burden in Bardet-Biedl syndrome: a survey of obesity and hyperphagia impacts [abstract]. *Hormone Res. Paediatr*. 2022;95(Suppl. 2):1-616.

Forsythe E, Mallya UG, Yang M, Huber C, Cala ML, Greatsinger A, Pomeroy J, Haqq AM. The multifaceted burden experienced by caregivers of individuals with Bardet-Biedl syndrome: findings from the CARE-BBS study [abstract]. *Hormone Res. Paediatr*. 2022;95(Suppl. 2):1-616.

Han JC, Reyes-Capo DP, Liu CY, Reynolds JC, Turkbey E, Turkbey IB, Bryant J, Marshall JD, Naggert JK, Gahl WA, Yanovski JA, Gunay-Aygun M. Comprehensive endocrine-metabolic evaluation of patients with Alström syndrome compared with BMI-matched controls. *J. Clin. Endocrinol. Metab*. 2018;103(7):2707-2719.

Haqq A, Chung W, Dolfus H, Iqbal A, Martos-Moreno G, Poitou C, Yanovski J, Malhotra S, Miller P, Yuan G, Forsythe E, Clement K, Argente J. Exploration of clinical improvements following setmelanotide in patients with Bardet-Biedl syndrome [abstract]. *Obesity* 2022;30(S1):55-293.

Harisseh R, Delale T, Yeh M, S Allas. SUN-609 livoletide (AZP-531), an unacylated ghrelin analogue, improves hyperphagia and food-related behaviors both in obese and non-obese people with Prader-Willi syndrome [abstract]. *J. Endocrine Soc.* 2020;4(Supplement_1): SUN-609.

Haws RM, Gordon G, Han JC, Yanovski JA, Yuan G, Stewart MW. The efficacy and safety of setmelanotide in individuals with Bardet-Biedl syndrome or Alström syndrome: phase 3 trial design. *Contemp. Clin. Trials Commun*. 2021;22:100780.

Haws R, Pomeroy J, Mallya U, Buckley B, Faucher A, Kyle R. Substantial burden associated with hyperphagia and obesity in children with Bardet-Biedl syndrome [abstract]. *Obesity*. 2022;30(S1): 55-293.

Hollander E, Levine KG, Ferretti CJ, Freeman K, Doernberg E, Desilva N, Taylor BP. Intranasal oxytocin versus placebo for hyperphagia and repetitive behaviors in children with Prader-Willi Syndrome: a randomized controlled pilot trial. *J. Psychiatr. Res*. 2021;137:643-651.

Holsen LM, Savage CR, Martin LE, Bruce AS, Lepping RJ, Ko E, Brooks WM, Butler MG, Zarcone JR, Goldstein JM. Importance of reward and prefrontal circuitry in hunger and satiety: Prader-Willi syndrome vs simple obesity. *Int. J. Obes. (Lond).* 2012;36(5):638-647.

Hor A, Richens R, Toth K, D’Silva S, Dodds J, Loughnan G, Markovic T, Campbell L, Viardot A. Long-acting GLP-1 agonist treatment in Prader-Willi syndrome: benefits on appetite, behaviour and cognition versus risks? Presented at: 10th International Prader-Willi Syndrome Organisation Concerence; November 13-17, 2019; Havana, Cuba.

Jacob R, Drapeau V, Tremblay A, Provencher V, Bouchard C, Pérusse L. The role of eating behavior traits in mediating genetic susceptibility to obesity. *Am. J. Clin. Nutr*. 2018;108(3):445-452.

Kayadjanian N, Vrana-Diaz C, Bohonowych J, Strong TV, Morin J, Potvin D, L Schwartz. Characteristics and relationship between hyperphagia, anxiety, behavioral challenges and caregiver burden in Prader-Willi syndrome. *PLoS One*. 2021;16(3):e0248739.

Kimonis V, Surampalli A, Wencel M, Gold JA, Cowen NM. A randomized pilot efficacy and safety trial of diazoxide choline controlled-release in patients with Prader-Willi syndrome. *PLoS One*. 2019;14(9):e0221615.

Kühnen P, Wabitsch M, von Schnurbein J, Chirila C, Mallya UG, Callahan P, Gnanasakthy A, Poitou C, Krabusch PM, Stewart M, Clément K. Quality of life outcomes in two phase 3 trials of setmelanotide in patients with obesity due to LEPR or POMC deficiency. *Orphanet J. Rare Dis*. 2022;17(1):38.

Kuppens RJ, Donze SH, Hokken-Koelega AC. Promising effects of oxytocin on social and food-related behaviour in young children with Prader-Willi syndrome: a randomized, double-blind, controlled crossover trial. *Clin. Endocrinol. (Oxf).* 2016;85(6):979-987.

Kweh F. Polymorphisms in the oxytocin receptor (OXTR) modulate response to intrasnasal oxytocin therapy in individuals with Prader-Willi syndrome. Presented at: PWS Research Symposium; October 4, 2018; Las Vegas, NV.

Lester S, Holland T, Gold JA, Leong V. Investigating the allocation of visual attention to salient stimuli in infants and young children with Prader-Willi syndrome. Poster presented at: 10th International Prader-Willi Syndrome Organisation Conference; November 13-17, 2019; Havana, Cuba.

Licenziati MR, Bacchini D, Crinò A, Grugni G, Fintini D, Osimani S, Ragusa L, Sacco M, Iughetti L, De Sanctis L, Franzese A, Wasniewska MG, Faienza MF, Delvecchio M, Esposito C, Valerio G. The Hyperphagia Questionnaire: insights from a multicentric validation study in individuals with Prader Willi syndrome. *Front. Pediatr*. 2022;10:829486.

Lundgren JD, Patrician TM, Breslin FJ, Martin LE, Donnelly JE, Savage CR. Evening hyperphagia and food motivation: a preliminary study of neural mechanisms. *Eat. Behav*. 2013;14(4):447-450.

Matesevac, L. PATH for PWS Study: A non-interventional, observational, natural history study of serious medical events in Prader-Willi syndrome. Presented at: FPWR Research Symposium & Family Conference; October3, 2019; New Orleans, LA.

McCandless SE, Yanovski JA, Miller J, Fu C, Bird LM, Salehi P, Chan CL, Stafford D, Abuzzahab MJ, Viskochil D, Barlow SE, Angulo M, Myers SE, Whitman BY, Styne D, Roof E, Dykens EM, Scheimann AO, Malloy J, Zhuang D, Taylor K, Hughes TE, Kim DD, Butler MG. Effects of MetAP2 inhibition on hyperphagia and body weight in Prader-Willi syndrome: A randomized, double-blind, placebo-controlled trial. *Diabetes Obes. Metab*. 2017;19(12):1751-1761.

Melo MCA, de Oliveira Ribeiro M, de Araújo CFC, de Mesquita LMF, de Bruin PFC, de Bruin VMS. Night eating in bipolar disorder. *Sleep Med.* 2018;48:49-52.

Miller JL, Tamura R, Butler MG, Kimonis V, Sulsona C, Gold JA, Driscoll DJ. Oxytocin treatment in children with Prader-Willi syndrome: a double-blind, placebo-controlled, crossover study. *Am. J. Med. Genet. A.* 2017;173(5):1243-1250.

NCT01818921. An efficacy, safety, and pharmacokinetics study of beloranib in obese subjects with Prader-Willi syndrome. <https://clinicaltrials.gov/study/NCT01818921>. 2013.

NCT02011360. Prader-Willi syndrome macronutrient study. <https://clinicaltrials.gov/study/NCT02011360>. 2016.

NCT02311673. Phase 2 trial to evaluate safety and efficacy of setmelanotide (RM-493) in obese participants with Prader-Willi syndrome. <https://classic.clinicaltrials.gov/ct2/show/NCT02311673>. 2016.

NCT03114371. Oxytocin intranasal administrations in children with Prader-Willi syndrome aged from 3 to 12 years. <https://clinicaltrials.gov/study/NCT03114371>. 2019.

NCT03274856. A study of GLWL-01 in patients with Prader-Willi syndrome. <https://clinicaltrials.gov/study/NCT03274856>. 2019.

NCT03440814. A study of diazoxide choline in patients with Prader-Willi syndrome. <https://classic.clinicaltrials.gov/ct2/show/NCT03440814>. 2020.

NCT03548480. Targeting the gut microbiome for Prader-Willi syndrome treatment. <https://clinicaltrials.gov/study/NCT03548480>. 2019.

NCT04966741. Setmelanotide in pediatric patients with rare genetic diseases of obesity. <https://clinicaltrials.gov/study/NCT04966741>. 2023.

NCT05044000. Effect of deep propioceptive stimulation in Prader-Willi syndrome. <https://classic.clinicaltrials.gov/ct2/show/NCT05044000>. 2023.

NCT05093634. EMANATE: A study of setmelanotide in patients with specific gene variants in the MC4R pathway. <https://www.clinicaltrials.gov/study/NCT05093634>. 2023.

NCT05541003. Understanding the role of gut microbiota in hyperphagia in Prader-Willi syndrome. <https://www.clinicaltrials.gov/study/NCT05541003>. 2024.

NCT05939453. Impact of bright light therapy on Prader-Willi syndrome. <https://classic.clinicaltrials.gov/ct2/show/NCT05939453>. 2023.

Nolan LJ, Geliebter A. Night eating is associated with emotional and external eating in college students. *Eat. Behav*. 2012;13(3):202-206.

Pedemonti B, Ceccomancini R, D'Acunti A, Stegmann J. Effectiveness of a transdisciplinary approach on hyperphagia management among patients with Prader Willi syndrome. *Endocrinol. Diabetes Nutr. (Engl Ed)*. 2023;70(5):347-351.

Provencher V, Pérusse L, Bouchard L, Drapeau V, Bouchard C, Rice T, Rao DC, Tremblay A, Després JP, Lemieux S. Familial resemblance in eating behaviors in men and women from the Quebec Family Study. *Obes. Res*. 2005;13(9):1624-1629.

Ryman D, Deal CL. Topline results of the CARE-PWS phase 3 study: intranasal carbetocin improves hyperphagia and anxiety and distress symptoms in Prader-Willi syndrome (PWS) [abstract]. *J. Endocrine Soc*. 2021;5(Supplement_1): A689-A689.

Salehi P, Hsu I, Azen CG, Mittelman SD, Geffner ME, Jeandron D. Effects of exenatide on weight and appetite in overweight adolescents and young adults with Prader-Willi syndrome. *Pediatr. Obes*. 2017;12(3):221-228.

Sherafat-Kazemzadeh R, Ivey L, Kahn SR, Sapp JC, Hicks MD, Kim RC, Krause AJ, Shomaker LB, Biesecker LG, Han JC, Yanovski JA. Hyperphagia among patients with Bardet-Biedl syndrome. *Pediatr. Obes*. 2013;8(5):e64-67.

Torok D, Kolouskova S, Walter P, Edsberg B, Dvorak RV. Tesomet - a new treatment opportunity in Prader Willi Syndrome. Results from Phase 2a exploratory studies in adult and adolescent patients. Presented at: 10th International Prader-Willi Syndrome Organisation Conference; November 13-17, 2019; Havana, Cuba.

Tsai JH, Crossnohere NL, Strong T, Bridges JFP. Measuring meaningful benefit-risk tradeoffs to promote patient-focused drug development in Prader-Willi syndrome: a discrete-choice experiment. *MDM Policy Pract*. 2021;6(2):23814683211039457.

van Santen HM, van Schaik J, IMAA van Roessel, J Beckhaus, S Boekhoff, Müller HL. Diagnostic criteria for the hypothalamic syndrome in childhood. *Eur. J. Endocrinol.* 2023;188(2):214-225.

Vander Wal JS, Gang CH, Griffing GT, Gadde KM. Escitalopram for treatment of night eating syndrome: a 12-week, randomized, placebo-controlled trial. *J. Clin. Psychopharmacol*. 2012;32(3):341-345.

Wabitsch M, Fehnel S, Mallya UG, Sluga-O’Callaghan M, Richardson D, Price M, P Kühnen. Understanding the patient experience of hunger and improved quality of life with setmelanotide treatment in POMC and LEPR deficiencies. *Adv. Ther*. 2022;39(4):1772-1783.

Yahia N, Brown C, Potter S, Szymanski H, Smith K, Pringle L, Herman C, Uribe M, Fu Z, Chung M, Geliebter A. Night eating syndrome and its association with weight status, physical activity, eating habits, smoking status, and sleep patterns among college students. *Eat. Weight Disord*. 2017;22(3):421-433.

Zorn S, von Schnurbein J, Schirmer M, Kohlsdorf K, Brandt S, Wabitsch M. Assessment of hyperphagia in patients with monogenic obesity. Poster presented at: the ESPE Annual Meeting; September 22-26, 2021; Virtual.

Zorn S, von Schnurbein J, Schirmer M, Brandt S, Wabitsch M. Measuring hyperphagia in patients with monogenic and syndromic obesity. *Appetite*. 2022;178:106161.
